# The relative importance of undesirable truths

**DOI:** 10.1007/s11019-012-9449-x

**Published:** 2012-11-19

**Authors:** Lisa Bortolotti

**Affiliations:** Philosophy Department, University of Birmingham, Edgbaston, B15 2TT UK

**Keywords:** Right not to know, Genetic testing, Personal narratives, Autonomy, Open future, Self-authorship, Self-knowledge

## Abstract

The right not to know is often defended on the basis of the principle of respect for personal autonomy. If I choose not to acquire personal information that impacts on my future prospects, such a choice should be respected, because I should be able to decide whether to access information about myself and how to use it. But, according to the incoherence objection to the right not to know in the context of genetic testing, the choice not to acquire genetic information undermines the capacity for autonomous decision making. The claim is that it is incoherent to defend a choice that is inimical to autonomy by appealing to autonomy. In this paper, I suggest that the choice not to know in the context of genetic testing does not undermine self-authorship, which is a key aspect of autonomous decision making. In the light of this, the incoherence objection to the right not to know seems less compelling.

## The incoherence objection to the right not to know

Consider the following case. Francis and his son George may have inherited the gene for Huntington disease. Whereas George wants to know whether he has the gene for the disease, Francis prefers to live his life without knowing. The problem is that, if George decides to be tested and results positive, then this means that Francis also has the gene. It would be difficult for George to keep the information from his father, so George’s wish to know is in conflict with Francis’s wish not to know. Does Francis have *a right not to know*?^1^ If so, where does this right come from?

Scenarios such as the conflict of interests between George and Francis illustrate the potential impact that a right not to know could have on people’s healthcare options, on their life projects, and even on their relationships with genetically related family members. That is why the choice not to know is so widely discussed in the philosophical literature, and a lively debate centres on whether such a choice should be safeguarded as a right (see for instance, Solbakk et al. 2009; Haÿry et al. 2007; Chadwick et al. 1997; Räikkä 1998).

The main justification for the right not to know is that one should be able to exercise at least some control over access to information about one’s health conditions and prospects, and that one’s desire not to know whether one will develop a certain condition should be respected. These considerations are usually grounded in the principle of respect for personal autonomy (e.g., Andorno 2004; Human Genetics Commission 2000, p. 19). But it has been argued that it is a mistake to defend the right not to know on the basis of personal autonomy, especially in the context of genetic testing.

One argument is that the choice not to know cannot always be justified on the basis of the principle of the autonomy of the individual, because the information obtained via genetic testing does not always belong to an individual alone. In the scenario above, we saw that if George had the gene for Huntington disease, then Francis would too. Francis does not want to know whether he has the gene, but George does. When individuals have conflicting interests and preferences, and information disclosed to one cannot be easily kept from the other, it is hard to respect the interests and preferences of both at once. Genetic information is typically shared, and an examination of its characteristics may invite a revision of our ethical framework which is based on the autonomy of the person as a self-standing individual.

Genetics supports a relational understanding of the person and therefore that genetic ethics requires ethical models which respect both individuals and groups (Widdows 2009, p. 173).

Another argument is that it is incoherent to justify the choice not to know on the basis of the principle of personal autonomy, because not knowing undermines the capacity to make autonomous choices. Consider our initial scenario. George wants to know whether he has the gene for Huntington disease, and if he obtains this knowledge, his future decisions will be informed by it. Francis prefers not to know and, if he manages to remain in ignorance about the possibility of developing Huntington disease, his future decisions will not be affected. According to the incoherence objection, George’s and Francis’s individual preferences are not neutral with respect to autonomy: George does not compromise the autonomy of his future decision-making, whereas Francis does.

Different versions of this objection have been put forward (see Harris and Keywood 2001; Malpas 2005; Rhodes 1998). Here are the key formulations:

[W]e cannot defend a right not to know our genetic information in the name of autonomy alone. […] Autonomy […] demands that we exercise our capacity to reason and this surely entails the pursuit of pertinent genetic information not the rejection of it. (Malpas 2005)

Now if autonomy is the ground for my right to determine my own course, it cannot also be the ground for not determining my own course. If autonomy determines my right to knowledge, it cannot also justify my refusing to be informed. I may not be aware of the moral implications of ceding autonomy by insisting on genetic ignorance, but the ramifications are there, nevertheless. (Rhodes 1998, p. 18)

[W]here I give someone (against their will) reliable information about themselves or their condition which is relevant to decisions they must make I may violate a liberty they assert but I do not violate their autonomy, for the information I give them is *necessary* for their autonomous decision making. (Harris and Keywood 2001, page 419, my emphasis)

In the rest of the paper I shall concentrate on the version of the incoherence objection put forward by John Harris and Kirsty Keywood, according to which knowledge of genetic information is necessary to the capacity for autonomous decision-making. The objection thus formulated invites us to reflect not only on the legitimacy of the right not to know, but also on what is valuable in the making of autonomous choices. It deserves attention because it has significant implications. If we accept this version of the incoherence objection, then we also have to accept that the choice of genetic ignorance^2^ is a choice that undermines the autonomy of future choices, and in this respect it is comparable to letting other people shape one’s life by selling oneself into slavery, or to taking drugs knowing that they will compromise one’s capacity to make rational decisions.

Contemporary studies in psychology and psychiatry support the general idea, frequently developed in leading philosophical theories of autonomy, that self-knowledge contributes to the exercise of personal autonomy in a way that promotes successful self-governance and well-being (see for instance, Wilson 2002). But the claim that self-knowledge enhances the quality of one’s autonomous choices is different from the claim that genetic information is necessary for autonomous decision making. First, it is not obvious that genetic information about oneself is the type of self-knowledge that contributes to the making of autonomous choices; and second, the nature of the contribution of genetic information to autonomous decision-making needs to be reviewed. Is such information really necessary for the exercise of autonomy?

In this paper I offer some reasons to resist the claim that makes the incoherence objection plausible, but I shall not offer any defence of the legitimacy of the right not to know, as I share the view defended by Widdows and by Harris and Keywood that one’s choice not to know should not be made into a right which trumps other morally relevant considerations. In “Autonomy as self-authorship”, I describe one central feature of the capacity for autonomous decision making, i.e. self-authorship. (In my interpretation, this is also the feature Harris and Keywood emphasise when they talk about the value of autonomy.) I suggest that self-authorship is not necessarily compromised by the choice of genetic ignorance. In “Not all knowledge about oneself is necessary to autonomy”, I compare the acquisition of genetic information with another form of knowledge that can be similarly distressing or unsettling, at least in the short term. This is knowledge of one’s biases in deliberation. I argue that information about one’s biases in deliberation necessarily contributes to self-authorship, whereas information resulting from genetic testing does not. My conclusion will be that choosing not to know information about oneself available through genetic testing is compatible with self-authorship, and thus the incoherence objection remains unconvincing.

## Autonomy as self-authorship

What we mean by autonomy is crucial to the debate about the right not to know and its justification. For instance, Matti Häyry and Tuija Takala argue that the perceived conflict between exercising autonomy and the choice not to know is legitimate only if we intend autonomy as the entitlement and the obligation to decide in accordance to the best information available (Häyry and Takala 2001, page 411). If information likely to be relevant to an agent’s decisions is withheld, then the agent cannot make fully informed decisions and autonomy is compromised. But if we mean something else by autonomy, for instance the entitlement to make decisions on whatever grounds the agent wishes, as long as she does not cause harm to others, then the choice not to know does not seem to conflict with the exercise of autonomy.

I want to develop Häyry and Takala’s insight that the notion of autonomy matters to an assessment of the incoherence objection. According to lay conceptions and recent philosophical accounts (e.g., Frankfurt 1988; Dworkin 1988; Moran 2001; Velleman 2006; McLeod 2002), autonomous agents are able to shape their future, within constraints set by their own personal limitations and their physical and social environment. They do so by developing a life plan which reflects their beliefs, desires and values. In some sense, for autonomous agents the future is *open* and can be *authored*.

The ability to choose our own life plan is arguably one of the essential conditions of the good life. What does this ability require? People must have cognitive and emotional skills that make them able to (a) compare (consciously or unconsciously) different life plans, (b) select one among those life plans they are able to consider, (c) transform this choice into the intention to behave in accordance with the chosen plan and (d) transform this intention into behaviour that actually conforms to the chosen option. Moreover, people must have skills that allow them to pursue different life plans with some definite chance of success, and they must be in a social context where these different life plans can actually be pursued. (Mameli 2007, p. 91)

One important aspect of this conception of autonomous agency is that people can become authors of their own life story, and this act of self-authorship is manifested (among other things) in the goals they set for themselves and in the choices they make. Agents pursue those goals and make those choices that are important to the type of person they are or they want to become, and that are largely consistent with their beliefs, desires, and values.

This is also the aspect of autonomy that Harris and Keywood (2001) focus on when they attack the legitimacy of a right not to know. They write:

The point of autonomy, the point of choosing and having the freedom to choose between competing conceptions of how, and indeed why, to live, is simply that it is only thus that our lives become in any real sense our own. The value of our lives is the value we give to our lives. And we do this, so far as this is possible at all, by shaping our lives for ourselves. Our own choices, decisions and preferences help to make us what we are, for each helps us to confirm and modify our own character and enables us to develop and to understand ourselves. (Harris and Keywood 2001, p. 420).

Obviously, knowledge of different sorts informs the making of autonomous choices. In a successful life plan goals are truly valued by an agent and are achievable, having being selected on the basis of an agent’s beliefs, desires and values, of her competencies and limitations, and of the relevant features of the surrounding physical and social environment. It is important for the agent to pursue her genuine interests and take into account existing constraints (Allport 1937; Ford 1992; Austin and Vancouver 1996; Armitage and Christian 2004; Kuhl and Beckman 1985). If the agent has a mistaken conception of what is important to her, she is likely to set for herself goals whose fulfilment will not make her happy. If she has an inflated conception of her own talents, she is likely to set for herself goals that are too ambitious, and this is likely to result in poor management of her resources and ultimately in failure and frustration. If other difficulties and potential obstacles are underestimated or ignored, the agent’s life plan may turn out to be unrealistic and may need to undergo a number of revisions and adjustments, which come with considerable psychological costs.

Information of the kind I have described certainly contributes to the making of rational life choices and to the fulfilment of one’s life goals, but should it be regarded as necessary to the capacity for autonomous decision making and self-governance? If an agent has an accurate understanding of her own potential and of the features of her environment that are relevant to the attainment of her goals, she will develop a life plan that is feasible and she will be more successful in pursuing her life goals. The aspirations of a woman who wishes to distinguish herself in the pursuit of science in a country where women are discouraged from going to university may be frustrated. Similarly, a very talented musician who does not realise how severe his arthritis is becoming may find that his dream of making a living as a performing pianist will not come true. The point I want to press with these examples is that personal information and information about the surrounding environment impinge significantly on the feasibility of life plans and on the likelihood of success, but is not necessary to the capacity most human agents have to shape their own lives. Failing to obtain such information does not rule out self-governance altogether.

There are forms of knowledge that are necessary to self-authorship, and a good candidate is knowledge of one’s own attitudes. Consider the following case. Gina learns that she suffers from a medical condition causing infertility, and this reduces the reproductive options readily available to her. She decides to start saving to be able to afford infertility treatment at a private clinic. Knowledge of her medical condition and of the options available to her does affect her decisions, and allows her to adjust her life plan to the newly discovered constraints. However, even if Gina had never discovered about her medical condition and its consequences, she would have preserved the capacity for rational decision making and self-governance. The difference would have been that her life plan would have had fewer chances to be fulfilled. Whether she discovers or remains ignorant about her chances to reproduce naturally, Gina engages in self-authorship by determining the best course of action given her attitudes and the constraints of the surrounding environment she is aware of.

Now let’s tweak the scenario. Suppose Gina has included motherhood in her life plan because, in the community where she lives, women of her age and status are expected to reproduce. If she examined her own beliefs, desires and values more closely, she would find that she does not have any strong desire to raise children, and that she is perfectly fulfilled by her successful career and her happy relationship with her husband. But people in her close social circle keep asking her why she does not want children, and she feels the pressure of their expectations. In this version of the scenario, Gina’s imperfect self-knowledge undermines the authorship of her choices. The choice to become a mother is not one that is genuinely supported by her attitudes, and her life plan does not reflect her genuine inclinations. We could describe the case differently and say that external pressure has conditioned and moulded her personal preferences to the point that she acquired an aspiration that she did not have to start with. But even so, it is likely that her aspiration to become a mother badly coheres with some of her other aspirations, thereby creating tensions in her life project overall. In this case, partial ignorance of her mind and lack of awareness of the external factors affecting her attitudes undermine Gina’s claim to self-authorship.

We need to ask now whether personal information obtained via genetic testing is the type of information that contributes to making more feasible life plans (such as the information Gina acquires about her infertility) or the type of information without which self-authorship and self-governance are compromised (such as Gina’s knowledge of her genuine attitude towards motherhood). Harris and Keywood argue for the latter option.

[A]bsence of crucial information is inimical to self government, to the ability to control one’s own destiny, and hence inimical to autonomy. Ignorance of crucial information is inimical to autonomy in a way that other autonomy-limiting choices are not. For where the individual is ignorant of information that bears upon rational life choices she is not in a position to be self-governing. If I lack information, for example about how long my life is likely to continue I cannot make rational plans for the rest of my life. If I do not know that my life is only likely to last five more years, rather than say twenty-five more, many of my priorities will be inappropriate and some will be self-defeating. (Harris and Keywood 2001, p. 421).

I agree with the authors that there is absence of information which is “inimical to self government”, but I do not think that the absence of genetic information fits this description. Why should an agent’s ignorance of her life expectancy compromise her capacity for rational decision making or self-governance? Her decisions and plans can be rational given the knowledge at her disposal, which does not routinely include information about when exactly she will die. More generally, agents shape their lives and pursue their goals in absence of complete and accurate information about their future health prospects and to consider them lacking in self-governance because of their partial ignorance is raising the bar too high.

Let’s go back to our initial case of genetic testing for Huntington disease. It is plausible that the reason why Francis does not want to know whether he has the gene for Huntington disease is that he does not want some options to be ruled out in advance by that information. He does not want his long-term plans to be shaped by the lingering fear of developing the disease (Bortolotti and Widdows 2011). George feels differently. He welcomes the possibility of constructing his life plan in the light of information about his probable future state of health, and wants to start developing strategies that will help him face the difficulties ahead. This makes good sense, given that Huntington disease in particular does impair one’s capacity for rational decision making. Gaining information about the likelihood of developing a debilitating condition such as Huntington disease has an impact on the decisions an agent makes, as Gina learning that she is not likely to reproduce naturally leads her to develop new strategies to achieve her goal of becoming a mother. However, choosing ignorance of genetic information does not necessarily make one’s future choices less authentic or less genuinely authored—those choices can still be in tune with one’s beliefs, desires and values.

Rejecting genetic information (when available) does not necessarily compromise self-authorship, which is a core feature of the capacity for autonomous decision making (or, as Harris and Keywood say, “the point of autonomy”, p. 420). Information about whether Francis and George have the gene for Huntington disease is relevant to *how* *open* their futures are and to whether they need to make contingency plans in order to achieve the goals they have set for themselves. However, rejecting such information does not make their future *closed* or *unauthored*. Francis and George preserve the capacity to shape their future by setting goals and making choices whether or not they decide to know about their likelihood to develop Huntington disease. The fact that they make different choices about whether to acquire genetic information is a manifestation of their different ways to shape their own future. One might find Francis’s way objectionable on the grounds that it does not allow him to develop contingency plans, but it seems far too strong to regard his choice as irrational, which we would be tempted to do if we were to embrace the incoherence objection.

## Not all knowledge about oneself is necessary to autonomy

I suggested that an agent’s knowledge that she may develop a disease such as Huntington does not impact on her capacity to write her own story, although it may contribute to changing the way in which the story is written. I also suggested there are forms of knowledge, that is, knowledge of the agent’s own mind and her awareness of the external factors that influence her attitudes, which have a more central role to play in enabling the making of autonomous choices and can even be regarded as necessary conditions for self-authorship. Reliable information about one’s own attitudes (beliefs, desires, values) is not just conducive to choices that are genuinely authored, but seems to be necessary for self-authorship.

One way in which the connection between self-knowledge and autonomy has been articulated in the recent philosophical and psychological literature is via the notion of self-narratives. Agents integrate significant episodes of their lives into a coherent story, a self-narrative, and recognise themselves in the leading character of the story they tell. The narrative is not a mere description or interpretation of the leading character’s attitudes and actions, but a way to impose coherence on life events, and to guide future behaviour on the basis of previously made commitments (Velleman 2006; McAdams 1997).

One thing a deliberate decision accomplishes, when it creates an intention, is to establish a constraint by which other preferences and decisions are to be guided. (Frankfurt 1988, page 175)

Some accounts of self-narratives focus on an individual conception of herself as an agent, but actual self-narratives are never developed in isolation from the social and physical environment in which the agent is embedded. Social expectations and cultural influences are reflected in the stories agents tell, and even in the plot which is generally structured around initial goals, partial achievements and set-backs, and final success. Stories are shaped not just by the agent’s understanding of her own beliefs, desires and values, but also by her social roles and relationships.

A claim that has received empirical support is that good self-narratives (that is, narratives that represent more or less accurately key events in one’s life and are largely coherent) contribute to unified agency and psychological well-being. When self-narratives diverge widely from reality or reflect a fragmented set of attitudes, then it is difficult for the agent to coordinate with other agents, to engage effectively with the surrounding environment and to develop a unifying sense of self and purpose which supports long-term planning. Self-narratives need to be shared. The social environment to which the agent is connected provides a useful feedback mechanism on the development of the narrative, and the views of friends and family help the agent inhabit an inter-subjective space where her representation of reality is constrained not just by reality itself but also by other people’s representations of it.

People with psychiatric disorders in which autobiographical memories or salience mechanisms are disrupted have been described as “unreliable autobiographers” (Gerrans 2009) and their success in developing and pursuing life projects has been questioned (Bortolotti et al. 2012). Correspondence between the self-narrative and significant life events prevents one from becoming isolated from one’s social context and detached from the surrounding environment. Reporting autobiographical facts inaccurately or unreliably does not yield cooperative communication and, as a result, alienates others. The person with a very idiosyncratic narrative may be avoided or ignored. Moreover, the gap between the narrative and reality engenders failed predictions and unsuccessful explanations of personal events, which compromise autonomous decision making.

In this framework, knowledge of the self matters to accurate and coherent narratives, and accurate and coherent narratives, in turn, matter to autonomous agency.^3^ Important decisions about the agent’s future are informed by her conception of her own attitudes and dispositions. By contributing to the construction of largely accurate and coherent self-narratives, knowledge of attitudes and dispositions becomes a core ingredient of self-authorship. Circumstances that allow people to learn about themselves in a way that can then inform their future decisions by enhancing self-authorship enable the exercise of personal autonomy and thus should be actively promoted. One good example of such a circumstance is when, talking to a good friend or even noting the casual comment of a stranger, one discovers something about one’s own attitudes or dispositions that one had not previously realised. When the new information brought to one’s attention is not a desirable one, this process of *self*-*revelation*–as Wilson (2002) calls it–may be upsetting at first, but is likely to have lasting benefits in the long run. Typically self-revelation occurs when one acquires self-knowledge not by introspection but by inference from how one’s behaviour is perceived or interpreted by others. As an extension of everyday forms of self-revelation, people can learn something new about themselves by participating in a psychological study (Bortolotti and Mameli 2006).

Consider the classic psychological study on obedience to authority (Milgram 1974). It may have been distressing for research participants to learn that they had been willing to inflict pain on other human beings for the mere reason that they were told to do so by someone in an apparent position of authority—this piece of information is likely to impact negatively on anybody’s self-conception and self-esteem. Yet, this insight into one’s behavioural dispositions potentially allows one to exercise better control on one’s future behaviour, as one is alerted to the fact that in certain situations one could easily be led to act against one’s own values. Similarly, in studies on how people make hiring decisions, research participants are likely to learn at debriefing that a variety of non-significant factors (gender, ethnicity, overall appearance, and sexual preference) impacted on how they assessed the performance of job candidates. For instance, it has been shown that, when qualifications and work experience are equivalent, overweight candidates are less likely to be selected, especially if they are female (Pingitore et al. 1994). Learning that one is disposed to discriminate against people who are overweight may be unsettling at first, as agents normally represent themselves in a positive light and are resistant to acknowledging that their beliefs or actions may be prejudiced. However, information learnt during one’s participation in a psychological study on prejudices in the job market can be taken into account when the next important hiring decision has to be made, to everybody’s advantage. Autonomy is promoted by letting agents make their own independent decisions, but also by ensuring agents have knowledge of the factors affecting their decisions (Bortolotti and Mameli 2006), especially when such factors can lead agents to make decisions that are not consistent with their explicit attitudes.

In the case of psychological experiments where agents learn something new and important about the factors influencing their decisions, the acquired self-knowledge can have a negative effect on self-esteem, but can also contribute significantly to making agents better at decision making, thus directly affecting their capacity to make autonomous choices that are authentic. Agents become aware of potential biases and have the opportunity to control the effects of such biases in future decisions. As a result, they can make decisions that are more attuned to their own beliefs, desires and values.

Is this form of self-knowledge, learning about one’s behavioural dispositions via reliable testimony or by participating in a psychological study, analogous to learning the results of genetic testing with respect to its contribution to autonomous decision making? We expect the acquisition of both types of knowledge to be potentially distressing. But in both cases the distress seems to be short-lived (Elms 1982; Kimmel 2001). Some research has been done on the psychological effects of genetic testing on people who are told that they may develop a disease such as breast cancer or Huntington disease. Predictably, people whose tests are positive tend to experience more distress than people whose tests are negative, but distress usually remains in the normal range and does not lead to either suicide or high levels of anxiety or depression (Marteau and Croyle 1998; Butow et al. 2003). When anxiety does increase as a result of the newly acquired information, this is usually only in the period immediately following the disclosure of the information (Lerman et al. 2002, page 793). One interesting result is that for some people knowing (even in case of positive results) turns out to be less distressing than living in uncertainty. In terms of psychological effects then, both coming to know about one’s biases in decision making and acquiring genetic information about oneself are potentially distressing, but have not been shown to be likely causes of severe anxiety or long-term depression.

What about analogies concerning the potential benefits of acquiring information? In general terms, both types of information have benefits. Acquiring genetic information, as Harris and Keywood emphasise and as I have already suggested, may allow an agent to revise and update her life plan and account for previously unforeseen contingencies (e.g. shorter life expectancy or declining cognitive capacities). Moreover, if anxiety is caused by uncertainty, then knowing helps reduce anxiety. But the acquisition of genetic information is unlikely to have the same type of authorship-enabling effects on decision making that knowledge of one’s biases in deliberation does have.

Knowledge that one’s judgement can be skewed or distorted by biases or prejudices that are not immediately accessible to introspection allows one to control the–often hidden–factors influencing one’s choices, and make choices that are better aligned with one’s attitudes. In other words, it prevents agents from making decisions (e.g. hiring a less qualified candidate because of gender biases) that would not be supported by, and would clash with, their own attitudes. Choosing genetic ignorance is not on a par with choosing to be sold into slavery or choosing to take drugs that compromise future decision making. This is because the acquisition of genetic information does not seem to play a significant role in helping agents to align their goals and their decisions with their attitudes. As previously suggested, the acquisition of genetic information may have implications for how open one’s future turns out to be. Such knowledge can inform the provision of contingency plans, and thus allow agents to continue to pursue their life projects when unforeseen difficulties and new constraints emerge; but it is not a necessary ingredient of self-authorship.

If this analysis is correct, and choosing genetic ignorance has no detrimental effect on autonomy as self-authorship, then it is not clear why it should be incoherent to appeal to autonomy in order to turn the choice not to know into a right. Moreover, arguments like the incoherence objection may have other undesirable consequences. They draw too much attention to genetic information at the expense of other forms of personal information that have the potential to make a greater contribution to self-authorship. And they could also blind us to individual differences in the way in which agents choose to manage the uncertainty that their future inevitably brings. That said, we need to acknowledge that the acquisition of personal information obtained via genetic testing is valuable even if it turns out not to be necessary for autonomous decision making. One of the reasons why such knowledge is indeed valuable lies in the importance of contingency planning. Knowledge of forthcoming adversities may not be necessary for autonomous decision making, but can help agents develop effective responses to such adversities and adjust their life plans accordingly. This requires resilience and creativity that are key and valuable features of human agency (Carel 2009; Bortolotti 2010).

## Conclusion

In this paper I discussed the incoherence objection which claims that the right not to know in the context of genetic information should not be grounded in autonomy because choosing not to know compromises autonomous decision making. I focused on one core aspect of autonomous decision making, what I called ‘self-authorship’. Agents can shape their own future by setting goals and making choices that fit their attitudes and thus become the people they want to be. I argued that choosing genetic ignorance does not necessarily prevent agents from exercising self-authorship.

## Footnotes

1 This example is taken freely from *The Exploratorium*, a resource of UC Berkeley Program in Genetic Counseling and Georgetown University Medical Center.

2 In this paper, by the expression ‘genetic ignorance’ I mean the rejection of personal information obtained via genetic testing.

3 Kennett and Matthews (2009) make this point with respect to psychiatric disorders affecting memory.
